# Supplementary material for: Dietary fatty acid metabolism of brown adipose tissue in cold-acclimated men
Source: Nat Commun. 2017 Jan 30;8:14146. doi: 10.1038/ncomms14146 (PMC5290270; doi:10.1038/ncomms14146)
Supplement: Supplementary Information — Supplementary Figures [file ncomms14146-s1.pdf]

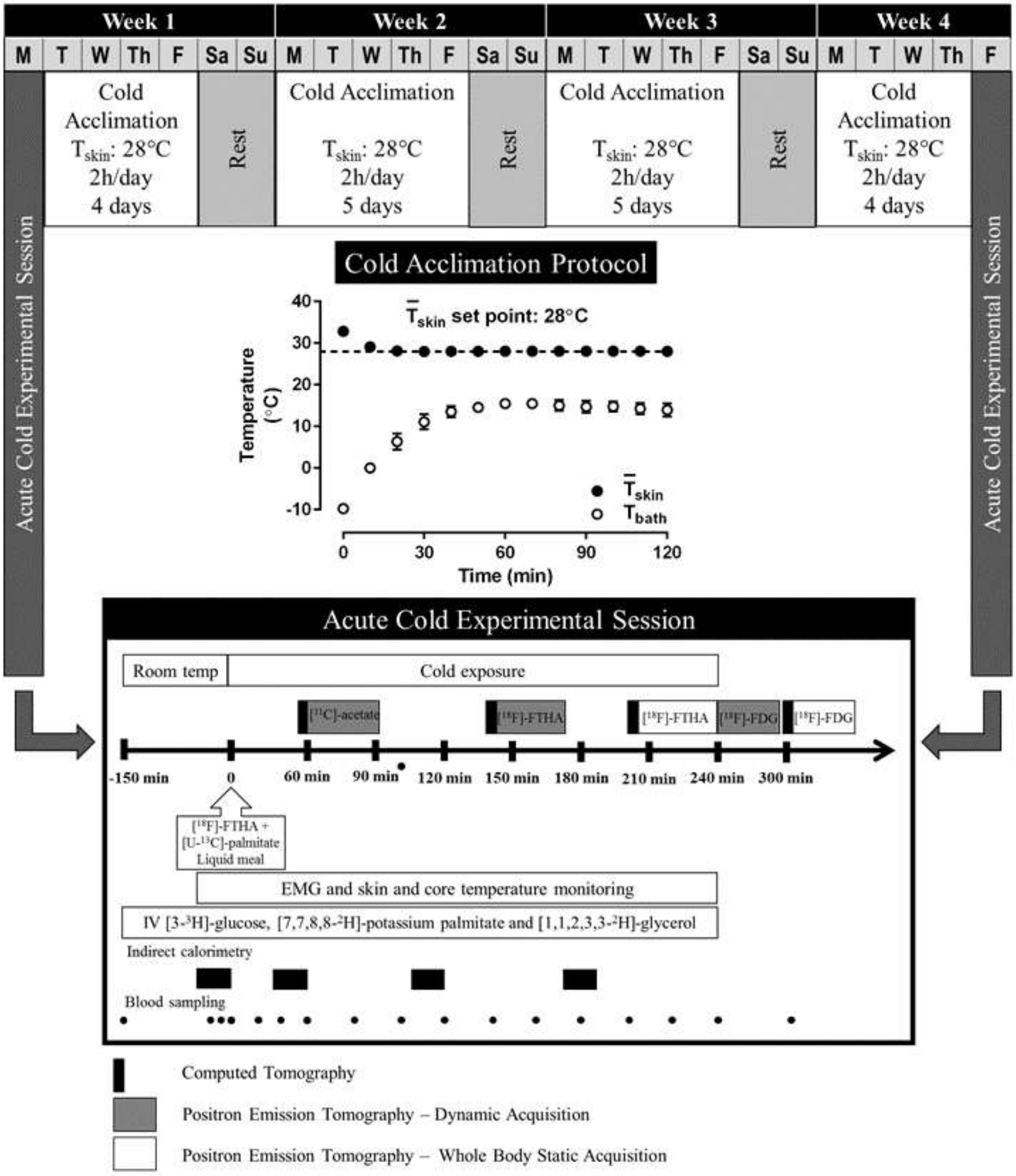

Supplementary Figure 1. Study protocol. Mean skin (closed circles) and inlet water temperature (open circles) of liquid-conditioned garment during typical cold acclimation session (N = 1), presented below timeline.

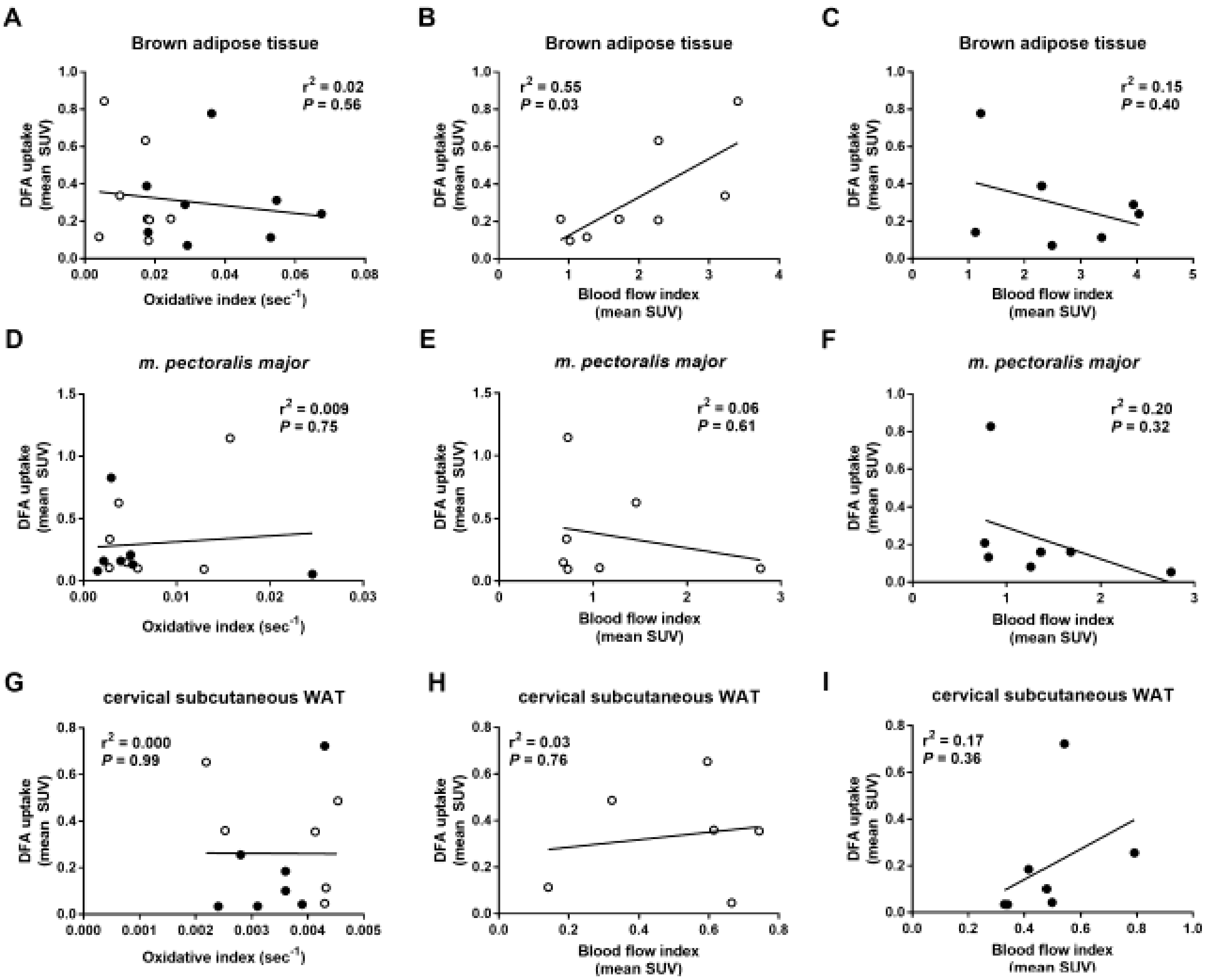

Supplementary Figure 2. Association between BAT dietary fatty acid uptake and BAT oxidative index (**A**) and BAT blood flow index pre-acclimation (**B**) and post-acclimation (**C**). Association between *m. pectoralis major* dietary fatty acid uptake and *m. pectoralis major* oxidative index (**D**) and *m. pectoralis major* blood flow index pre-acclimation (**E**) and post-acclimation (**F**). Association between cervical subcutaneous WAT dietary fatty acid uptake and cervical subcutaneous WAT oxidative index (**G**) and cervical subcutaneous WAT blood flow index pre-acclimation (**H**) and post-acclimation (**I**) (n= 8).

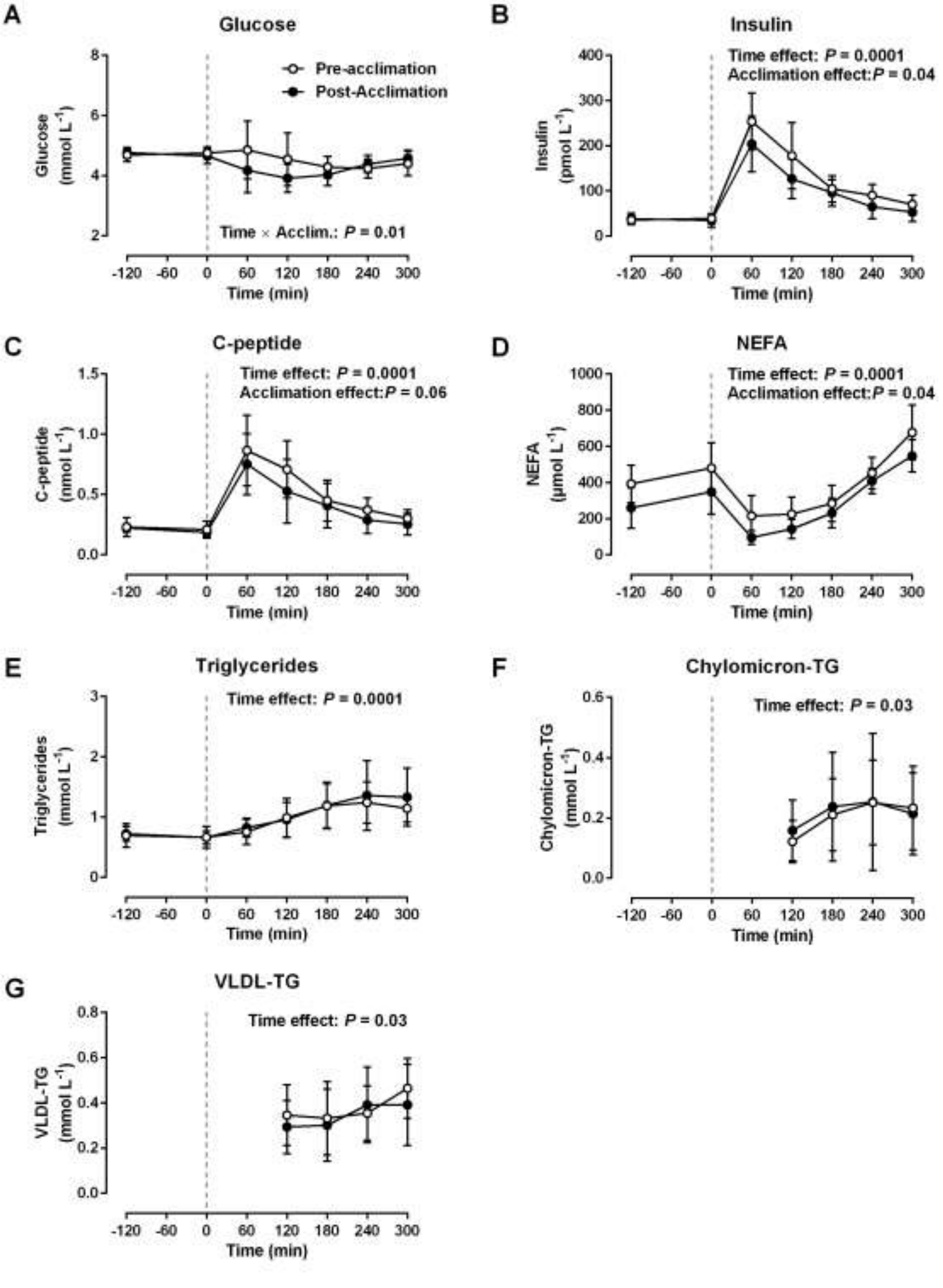

Supplementary Figure 3. Plasma glucose (**A**), insulin (**B**), C-peptide (**C**), NEFA (**D**), total TG (**E**), chdylomicron-TG (**F**) and VLDL-TG (**G**) after standard liquid meal intake at time 0, pre- (open circles) and post-acclimation (closed circles). Data are reported as the mean with 95% CI, n = 8. Repeated measures two-way ANOVA with Bonferonni post-hoc test.

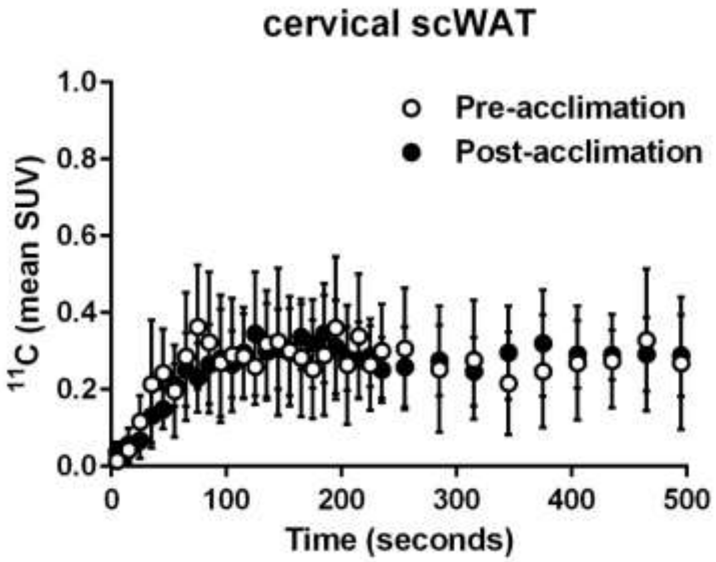

Supplementary Figure 4. ^{11}C time-radioactivity curves over the first 500 s of acquisition after [^{11}C]acetate injection before and after cold acclimation in cervical subcutaneous white adipose tissue. Data are reported as the mean with 95% CI, n = 8.
